# Long-Term Efficacy of Non-steroid Immunosuppressive Agents in Anti-Muscle-Specific Kinase Positive Myasthenia Gravis Patients: A Prospective Study

**DOI:** 10.3389/fneur.2022.877895

**Published:** 2022-06-13

**Authors:** Ying Tan, Jiayu Shi, Yangyu Huang, Ke Li, Jingwen Yan, Li Zhu, Yuzhou Guan, Liying Cui

**Affiliations:** ^1^Department of Neurology, Peking Union Medical College Hospital, Chinese Academy of Medical Sciences, Beijing, China; ^2^Department of Nuclear Medicine, Peking Union Medical College Hospital, Chinese Academy of Medical Science, Beijing, China

**Keywords:** myasthenia gravis, minimal manifestation status, autoimmune, anti-AChR antibody, anti-MuSK antibody

## Abstract

**Background and Purpose:**

Anti-muscle-specific kinase (MuSK) positive myasthenia gravis (MG) is characterized by a high relapsing rate, thus, choosing the appropriate oral drug regimen is a challenge. This study aimed to evaluate the efficacy of oral immunosuppressants (IS) in preventing relapse in MuSK-MG.

**Methods:**

This prospective cohort observational study included patients with MuSK-MG at Peking Union Medical College Hospital between January 1, 2018, and November 15, 2021. The patients were divided into 2 groups: those with (IS+) or without (IS-) non-steroid immunosuppressive agents. The primary outcome was relapsed at follow-up, and the log-rank test was used to compare the proportion of maintenance-free relapse between the groups; hazard ratio (HR) was calculated using the Cox proportional hazards models.

**Results:**

Fifty-three of 59 patients with MuSK-MG were included in the cohort, 14 were in the IS+ group, and 39 were in the IS- group. Twenty-four cases in the cohort experienced relapse at least once; the relapse rate was 2/14 (14.3%) in the IS+ group and 22/39 (56.4%) in the IS- group. At the end of follow-up, the proportion of maintenance-free relapse was significantly different between the two groups (log-rank χ^2^ = 4.94, *P* = 0.02). Of all the potential confounders, only the use of IS was associated with a reduced risk of relapse. The HR for relapse among patients in the IS+ group was 0.21 (95%CI 0.05–0.58) and was 0.23 (95%CI 0.05–0.93) in a model adjusted for age, sex, relapse history, highest Myasthenia Gravis Foundation of America (MGFA), and accumulated time of steroid therapy.

**Conclusions:**

This study provides evidence that oral non-steroid immunosuppressive agents may be beneficial in reducing relapse in patients with MuSK-MG.

## Introduction

Myasthenia gravis (MG) is caused by antibodies directed against the acetylcholine receptor (AChR), or other structural proteins of the neuromuscular junction. In 2001, 70% of AchR-Ab-seronegative MG patients were discovered positive in antibodies against muscle-specific kinase (MuSK) (1). The activation of MuSK, anchored in skeletal muscle, is responsible for the clustering of AChR at the neuromuscular junction (2). Patients with MuSK antibody-positive MG often have facial, neck, and respiratory weakness, but they have less prominent ocular findings compared with AchR antibody-positive MG.

The anti-MuSK subtype of MG presents a different response to immunomodulatory regimens compared to AchR MG, the proportion of patients with MuSK-MG requiring high doses and prolonged treatment to achieve full control of the disease seems to be higher (3–6). In most instances, patients with MuSK-MG respond to immunosuppressants (IS). Steroid, azathioprine (AZA), tacrolimus (TAC), mycophenolate mofetil (MMF), cyclosporine A (CsA), methotrexate (MTX), and rituximab (RTX) have been tried with success in patients with MuSK-MG and patients with AchR-MG (7–14). However, there is a lack of prospective data and a large sample to verify the effect of using oral non-steroid IS in MuSK-MG. Furthermore, previous studies did not consider exacerbation as a primary endpoint, and information about relapse is lacking (15, 16). Here, we conducted a prospective observational cohort study in Chinese patients with MuSK antibody-positive MG to determine the association between relapse risk and the use of oral non-steroid IS.

## Patients and Methods

### Participants

Patients were identified through the Peking Union Medical College Hospital (PUMCH) MG registry platform. The study was approved by the regional ethics committee of PUMCH, and participants provided written consent to registration in the MG registry and the use of recorded data for research purposes. The study was conducted from January 1, 2016 to November 30, 2021. This study followed the Strengthening the Reporting of Observational Studies in Epidemiology (STROBE) reporting guideline for cohort studies.

All patients included were diagnosed with MG. We prospectively collected demographic information and data on the course of illness, medication, neurological physical examinations, MGFA classification, MGC, MG-ADL scores, and Repetitive Nerve Stimulation (RNS) results. Patients' serum samples were acquired prospectively at enrolment. Serum AChR antibody titers and MuSK antibody were estimated by the immunoprecipitation methods using ^125^I-alpha-bungarotoxin and ^125^I-MuSK, respectively (RSR Limited, Cardiff, UK). All MuSK antibody-positive patients with MG were enrolled in the cohort.

### Therapeutic Regimens

In this study, participating patients with MuSK-MG, either received or did not receive IS after a complete discussion with the neurologists. Financial burden and potential AEs are the main concerns for patients who refused IS. We defined the IS+ group as treatment with one or more non-steroid IS for at least 6 months and the IS- group as treatment with only steroids during the follow-up. The following exclusion criteria were applied: the duration of observation was < 6 months, < 2 follow-up visits, concurrent neurologic diseases interfering with assessment, and immunosuppressive therapy for other indications during the observation.

Non-steroid IS dosing range was as follows: AZA 100–150 mg/day, TAC 3–5 mg/day, and MMF 1,000–3,000 mg/day. In the long-term follow-up, specialists set the achievement of MMS and better as the treatment goal and reduced the steroids to the minimum maintenance dose according to the long-term side effects of the steroids. All IS+ and IS- patients were treated with rescue therapy after an exacerbation, including intravenous methylprednisolone pulse therapy, intravenous immunoglobin injections (IVIG), and plasma exchange (PLEX).

### Follow-Up and Outcome Measurements

Patients were followed up by the specialist group every 6 month. Medication, MGC score, MG-ADL score, MG-PIS classification, and MuSK antibody results were recorded at each follow-up visit. Drug side effects were regularly monitored.

The study's primary outcome was relapsed, defined as a physician-confirmed exacerbation of MG in a previously stable state, except for other possible contributors to the exacerbation of weakness, such as electrolyte disturbance, infection, etc. A general ΔMGC score of > or equal to 3, treatment with rescue therapy, and/or hospitalization was considered clinically significant (17). The interval between the first relapse and time 0 after enrolment was recorded in days. We also assessed whether the association between IS therapy and relapse differed between the following subgroups: age at disease onset, high *vs*. low MuSK antibody titer, onset type, high *vs*. low MGFA subtype, and with *vs*. without relapse history before enrollment.

### Sample Size Calculation

The primary endpoint of this study was to calculate whether the use of IS significantly reduced the risk of relapse, and the results were calculated according to Cox risk proportional model, with Power calculated based on reference to Rosner and Freedman et al. The relapse rate of MuSK-MG in the IS+ group was 15% according to Evoli et al. (6) and 56% according to Guptill et al. (15), in the IS- group treated with steroids alone. Assuming IS+ and IS- patients were divided in a 1:1 ratio, at an expected dropout rate of 5%, 26 patients were required in each group. Then, we further assumed a postulated hazard ratio (HR) of 0.25 and α (two-sided) of 0.05, this study had more than 80% power to detect an HR of 0.25 or lower.

### Statistical Analysis

Continuous variables that were not normally distributed were expressed as the median and interquartile range (median, IQR) and categorical variables were expressed as frequencies and percentages (%). The χ^2^ and Mann-Whitney U tests were used for the comparison of categorical and continuous variables that were not normally distributed between the IS+ and IS- groups, respectively. Group differences in relapse risk were assessed using Kaplan-Meier curves, while univariate and multivariate hazard ratios (HRs) were assessed from Cox proportional hazards regression. The association of IS application with relapse of MG was compared in each subgroup: gender, age of onset, first onset muscle group, highest MGFA, relapse before time 0, initial MuSK antibody titters, and different disease duration subgroups. Stratified Cox risk proportional regression models were applied for subgroup analysis. All statistical results were significant by taking an α <0.05, two-sided test. Prism 7 (GraphPad Software) was used for unadjusted statistical tests. SPSS 28.0 (IBM) was used for statistical analysis.

## Results

### Patient Characteristics

**Figure 1:**
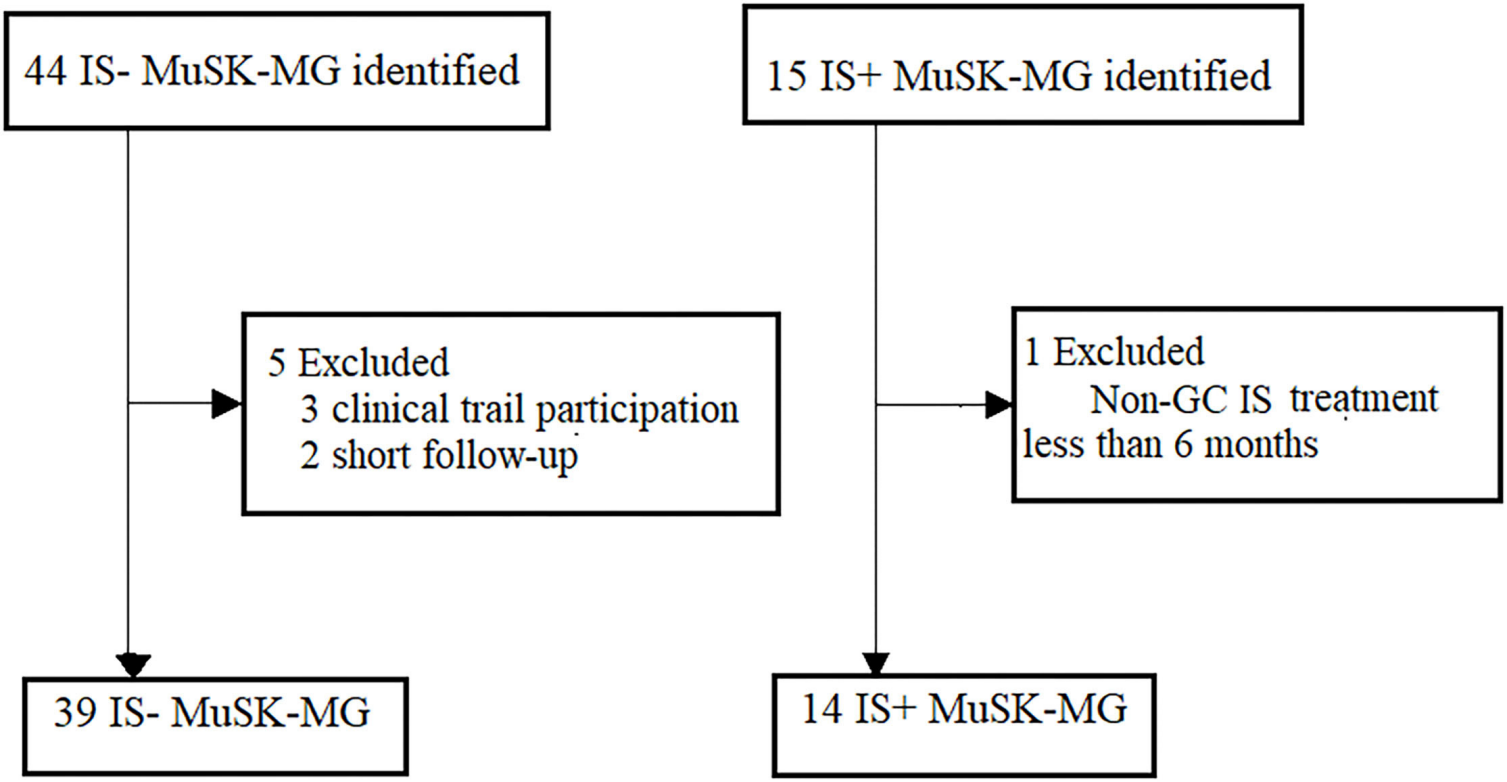
Recruitment of patients with MuSK-MG. Patient recruitment to the different treatment groups, respectively. MuSK, muscle-specific tyrosine kinase; non-GC IS, non-glucocorticoid immunosuppressants; IS+, immunosuppressants combined with prednisone therapy; IS-, prednisone monotherapy.

The study flowchart is shown in Figure 1. We identified 59 patients with MuSK antibody-positive in the database, 53 patients fulfilled the inclusion criteria and exclusion criteria (Figure 1). Of the 53 eligible patients, 39 patients (73.60%) were women; the median age of onset was 47 years (IQR 34–55). Fourteen of them received IS and steroid combination therapy [10 (71.40%) women]. The baseline characteristics of the two groups were largely comparable, including age at onset, gender composition, first muscle group affected, highest MGFA, severity as reflected by the most recent MGC/ADL score before time 0, MuSK antibody titers at time 0, RNS results, number of cases with muscle atrophy, or duration of disease before time 0 (Table 1A).

**Table 1A:** Baseline characteristics of MuSK-MG.

**Name**	**Results**		
	**Total**	**IS+ group**	**IS- group**	***P*** **-value**
Number of patients, *n*/total (%)	53/53 (100%)	14/53(26.42%)	39/53 (73.58%)	NA
Onset age^c^, y	47 (34, 55)	46.50 (28.50, 52.25)	44 (34, 53)	0.96^a^
Late onset (≥50), *n*/total (%)	20/53 (37.74%)	6/14 (42.86%)	13/39 (33.33%)	0.54^b^
Female, *n*/total, (%)	39/53 (73.58%)	10/14 (71.43%)	29/39 (74.36%)	0.53^b^
Information prior to time 0				
First onset muscles, *n*/total (%)				0.75^b^
Extraocular muscle	35/53	10/14	25/39	
Bulbar muscle or neck or facial muscle	12/53	2/14	10/39	
Limb or trunk muscle	6/53	2/14	4/39	
Disease duration, months	12 (6, 47)	35 (11.8, 69)	12 (4, 24)	0.05^a^
History of relapse, *n*/total (%)	16/53 (30.19%)	11/14 (78.57%)	5/39 (12.92%)	<0.01^b^
Steroid therapy, *n*/total (%)	24/53 (45.28)	10/14 (71.43%)	14/39 (35.90%)	0.03^b^
History of IS therapy, *n*/total (%)	6/53 (11.32%)	4/14 (28.57%)	2/39 (5.13%)	0.04^b^
IVIG within 3 months, *n*/total	22/53	5/14	17/39	0.74^b^
Information at time 0				
MGC score^c^	6 (3, 12)	4 (0, 12.75)	6 (3, 12)	0.42^a^
ADL score^c^	3 (2, 8)	2.5 (0, 9.5)	5 (2, 8)	0.39^a^
Muscle atrophy, *n*/total	6/53	3/14	3/39	0.21^b^
limb/facial muscle/lingual muscle, *n*	4/1/1	2/1/0	2/0/1	
MuSK-Ab^c^	1.13 (0.82, 1.45)	1.36 (0.83, 1.46)	1.07 (0.81, 1.43)	0.36^a^
RNS decrement, *n*/total (%)	38/51(74.5%)	11/14 (78.6%)	27/37(73.0%)	1.00^b^

a *Mann-Whitney U test;*

b *Fisher exact test;*

c *Described by Median (IQR)*.

Eleven (78.6%) and 5 (12.8%) patients had a history of MG relapse before enrolment in the two groups, with a statistically significant difference (*p* < 0.01). Six patients had a history of non-steroid IS therapy before enrolment, four in the IS+ group and two in the IS- group, two patients discontinued AZA treatment due to adverse events (AEs) before enrolment: 1 with drug-related granulocyte deficiency and another with an allergic reaction at the first week. The number of patients accepting steroid therapy at time 0 was 10/14 (71.4%) in the IS+ group and 14/39 (35.9%) in the IS- group, with statistically significant difference. The number of patients with a history of IVIG application within the 3 months prior to time 0 was 5 (35.7%) and 17 (43.6%) in the IS+ group and the IS- group, with no statistical difference (*p* = 0.74). One patient had undergone thymectomy, and the interval from surgery to enrolment was up to 4 years.

### Follow-Up Outcomes

The number of patients receiving steroids in the two groups was 14/14 cases (100%) and 36/39 cases (92.3%), respectively. The median daily dosage of steroids was comparable between the IS+ group and the IS- group at the end of follow-up (10 [9.38–15] mg and 10 [5–12], *p* = 0.40). The number of patients who successfully discontinued steroids was slightly greater in the IS- group (3/39) than that in the IS+ group (0/14), although the results were not statistically different (*p* = 0.09). The other three outcomes, including ADL score, MGC score, improving in ADL, and improvement in QMG, did not have statistical differences in the IS+ group and IS- group (Table 1B).

**Table 1B:** Follow up outcomes of MuSK-MG.

**Name**	**Results**		
	**Total**	**IS+ group**	**IS- group**	***P*** **-value**
Information after enrollment				
Highest MGFA classification, *n* (*n*/total)				0.65^b^
I	1 (1/53)	0	1 (1/39)	
II	30 (30/53)	7 (7/14)	23 (23/39)	
IIa/IIb, *n*	0/28	0/7	0/7	
III	10 (10/53)	4 (4/14)	6 (6/39)	
IIIa/IIIb, *n*	0/8	0/4	0/6	
IV	5 (5/53)	2 (2/14)	3 (3/39)	
IVa/IVb, *n*	0/4	0/2	0/1	
V	7 (7/53)	1 (1/14)	6 (6/39)	
Relapse, *n*/total (%)				0.01^b^
0	29/53 (54.72%)	12/14 (85.71%)	17/39 (43.59%)	
≥1	24/53 (45.28%)	2/14 (14.29%)	22/39 (56.41%)	
Gap from time0 to first relapse ^d^, days	0 (0, 420)	NA^c^	210 (0, 480)	NA
Final visit				
MGC score^d^	0 (0, 3)	0 (0, 3.50)	0 (0, 3)	0.63^a^
ADL score^d^	0 (0, 2)	0 (0, 2)	0 (0, 2)	0.56^a^
PIS classification, *n*/total (%)				0.66^b^
MM or better	30/53 (56.60%)	9/14 (64.29%)	21/39 (53.85%)	
Improved	17/53 (32.08%)	5/14 (35.71%)	12/39 (30.77%)	
Unchanged	1/53 (1.89%)	0	1/39 (2.56%)	
Worse	4/53 (7.55%)	0	4/39 (10.26%)	
Died	1/53 (1.89%)	0	1/39 (2.56%)	
Follow up time^d^, days	814 (540, 1110)	780 (352, 915)	840(630, 1290)	0.79^a^
Improving MGC^d^	3 (0, 9)	3 (0, 5.3)	3 (0, 11)	0.70^a^
Improving ADL^d^	2 (0, 5)	2 (0, 5.5)	2 (0, 6)	0.81^a^
Rate of steroid use, *n*/total (%)	50/53 (94.34%)	14/14 (100%)	36/39 (92.31%)	0.54^b^
Highest steroid dose, mg/d, *n*/total				0.02^b^
0	3/53	0	3/39	
1–20	0	0	0	
20–50	28/53	4/14	24/39	
>51	22/53	10/14	12/39	
Number of stop using steroid, *n*/total	8/53	0	8/39	0.09^b^
Steroid dose at last visit^d^, mg/d	10 (5,13.80)	10 (9.38, 15)	10 (5, 12)	0.41^a^
Accumulated time for steroid^d^, days	1020 (408.5, 1545)	1020 (414, 1507)	720 (390, 1470)	<0.01^a^

a *Mann-Whitney U test;*

b *Fisher exact test;*

c *Uncountable because of only two cases with relapsing events in IS+ group, 1 was at 570 days and 1 was at 1,080 days after enrollment;*

d *Described by Median (IQR)*.

Non-steroid IS medications included AZA (n = 9, 9/14, 48%) and TAC (n = 5, 5/14, 35.7%). No participants discontinued IS therapy owing to severe AEs during follow-up, thus, suggesting good tolerability. None of the patients was maintained on ChE-I at the end of the follow-up.

### Effects of Intervention

The median duration of observation was comparable between the IS+ group and the IS- group (780 [352, 915] days vs. 840 [630, 1290] days, *p* = 0.70). Log-rank tests did not reveal an association between relapse and factors, such as gender, initial symptoms, history of previous relapses, duration of disease before time 0, antibody titer at time 0, highest MGFA classification, and length of steroid use (Table 2).

**Table 2:** Log-Rank test of relapse and demographic characteristics.

	**Relapse**			
**Name**	**Median survival time**	**95%CI**		**Log-Rank *p*-value**
Type of sex				0.61
Male	1,080	306.71	1853.29	
Female	1,200	663.40	1736.61	
Onset age				0.03^*^
<50	660.00	384.48	935.52	
≥50	NA^a^			
Onset symptom				0.52
Extraocular muscle	1,080	535.57	1624.44	
Bulbar muscle or neck or facial muscle	810	0	1674.40	
Limb or trunk	NA^a^			
History of relapse				0.23
Yes	NA^a^			
No	1,080	716.20	1443.80	
Highest MGFA				0.55
I	1,080^b^	NA		
II	1,200	431.46	1968.54	
III	750	NA^a^		
IV	660	203.18	261.78	
V	810	345.36	133.10	
IS therapy				0.02^*^
IS+	NA^a^			
IS-	750	586.79	913.21	

* *With a significant difference;*

a *Uncountable because less than 50% of incidents occurred;*

b *only 1 patient in this group and taking the maximum survival time*.

**Figure 2:**
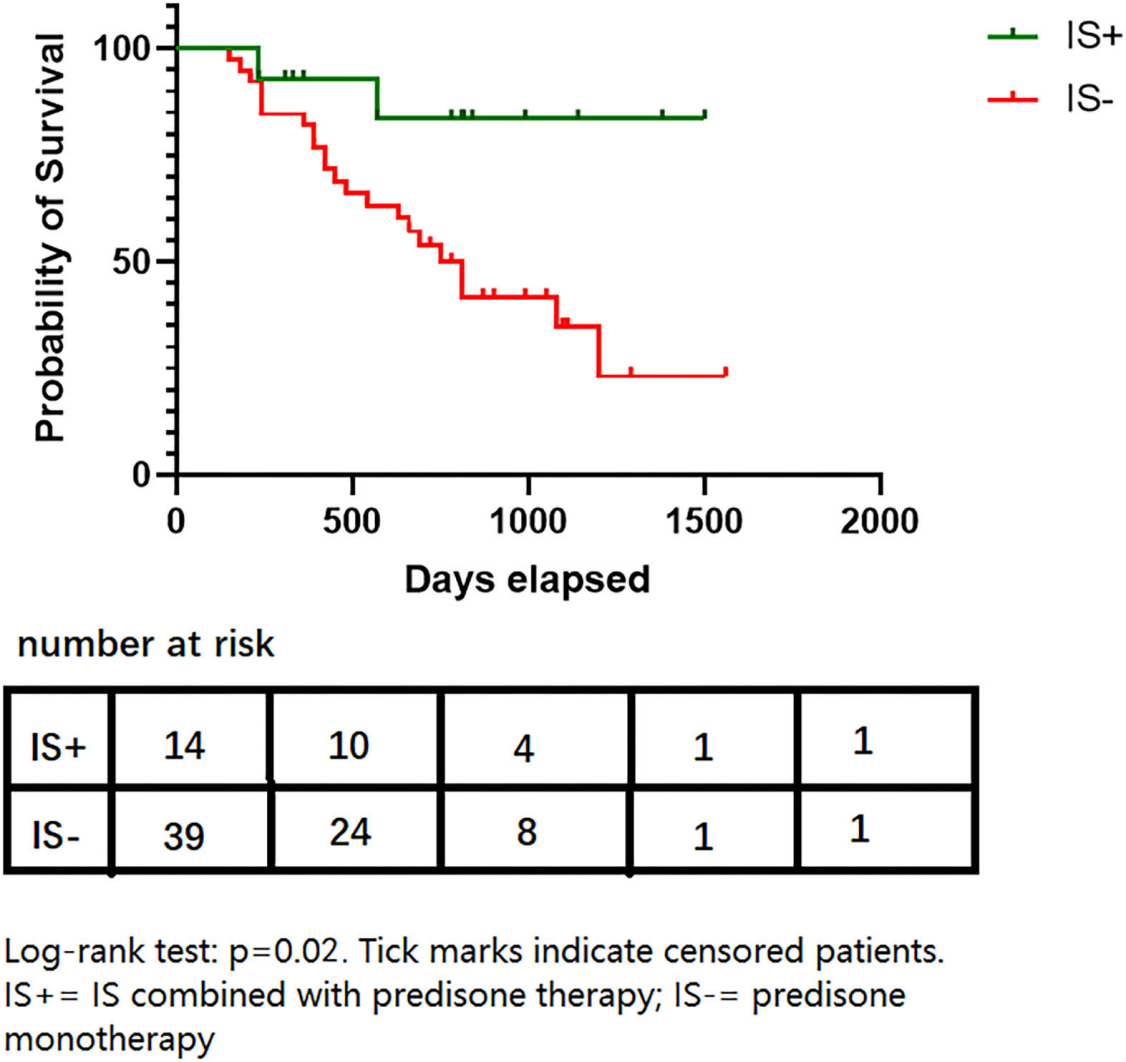
Survival proportions of two groups. Log-rank test: *p* = 0.02. Tick marks indicate censored patients. IS+, IS combined with prednisone therapy; IS-, prednisone monotherapy.

The IS was also associated with a longer duration of remission than steroid monotherapy (median 210 [0–480] days for steroid monotherapy; data not available for the IS+ group since 12/14 patients remained stable; HR = 0.21, 95% CI,0.05–0.58, *p* = 0.03, Figure 2). This association remained statistically significant after adjustment for age of onset, gender, relapse history, highest MGFA, and accumulation of steroid application (HR = 0.23, 95% CI,0.05–0.93, *p* = 0.04: Table 3). The proportion of patients in a clinically stable state at 500 and 1000 days was higher with IS than with steroid monotherapy (500 days: 14/14 [100%] IS treatment *vs*. 25/39 [64.1%] controls, *p* < 0.01; 1000 days: 13/14 [92.9%] and 15/39 [38.5%], *p* < 0.01).

**Table 3:** Cox regression^a^: analysis for IS therapy and MG relapses.

		**Unadjusted**		**Adjusted^b^**	
**Group**	**Relapse, *n*(%)**	**HR(95%CI)**	***P*** **-value**	**HR(95%CI)**	***P*** **-value**
IS+	2(14.3%)	0.21 (0.05–0.58)	0.03	0.23 (0.05–0.93)	0.04
IS-	22(56.4%)	1 (Reference)	NA	1 (Reference)	NA

a *Forward: LR;*

b *Correcting factors include age, gender, relapse history, highest MGFA, and accumulated time of steroid use*.

Log-rank test revealed a weak association between age and relapse, which disappeared after adjustment in the multivariable Cox regression model (*p* = 0.06). The patients were grouped according to antibody titer and a multiple Cox regression model showed an HR = 1.02, (95% CI, 0.43–2.43, *p* = 0.96) for relapse in the higher antibody titer group after adjustment. There was no statistical difference between the higher and the lower antibody titer group in age at onset, gender, duration of disease, steroid therapy and IS therapy before enrollment (Supplementary Table_e1).

### Subgroup Analysis

**Figure 3:**
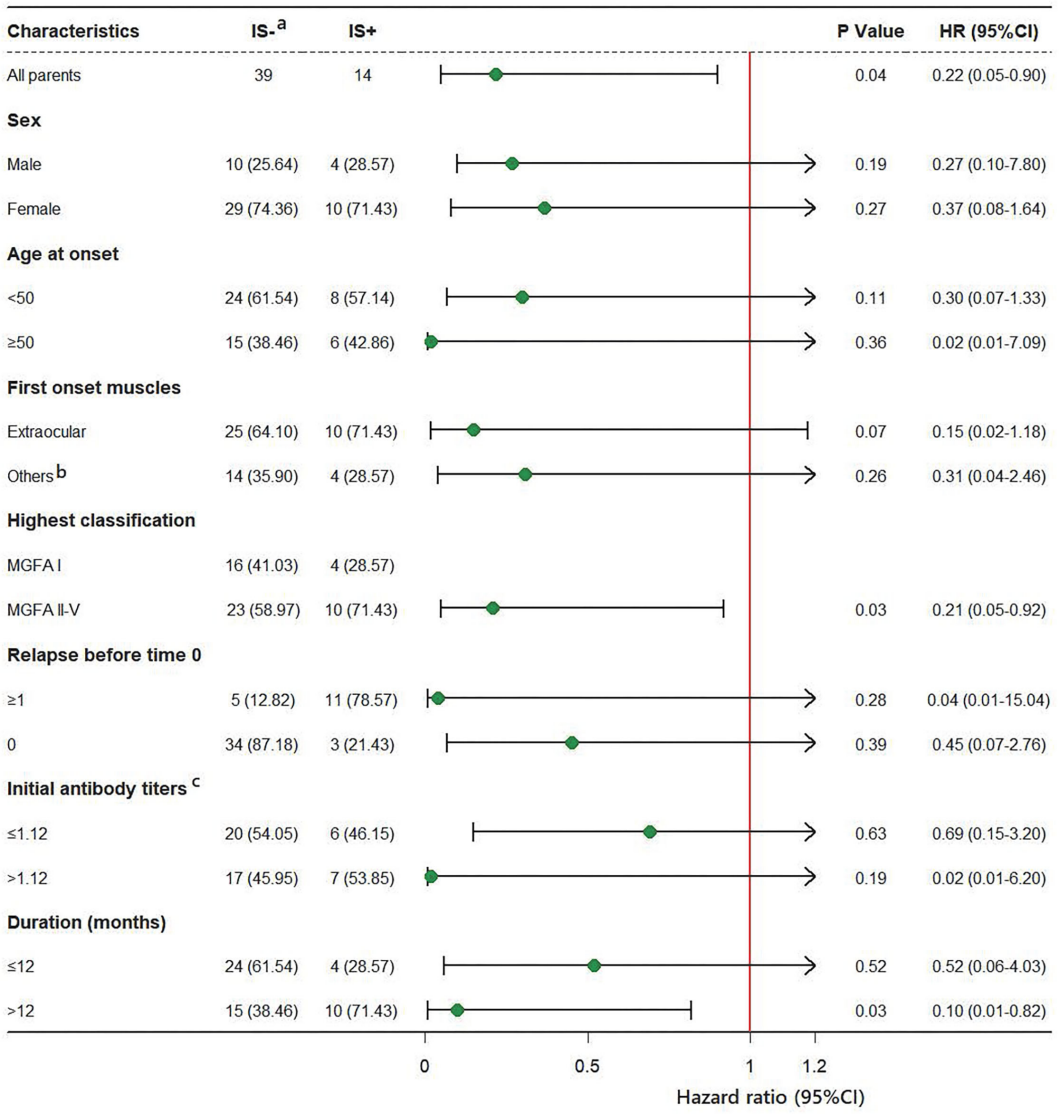
HRs for relapse in subgroup analysis. (a) Data of group IS- used as a reference. (b) Other muscle groups: bulbar muscles, facial muscles, limb muscles and neck muscles are involved. (c) Three missing data points.

Subgroup analyses are shown in Figure 3, in most of the subgroups, IS use was shown to correlate with a reduction in relapse events. Because of the small sample size in each subgroup, interaction analyses were not performed. IS showed better protection in subgroups with longer disease duration before time 0 (>12 months) and MGFA classifications II-V; however, it needs further study whether these results are statistically significant. Data analysis of other subgroups, such as gender, age at onset, different forms of onset, different relapse histories, and different antibody groups showed no association between IS and relapse.

## Discussion

In this prospective cohort study of MuSK-positive patients with MG, we observed that long-term IS treatment yielded a 78% reduction in relapse events with a median follow-up of 816 (540, 1110) days. The most prominent AEs were granulocytopenia (AZA-related, 1 case), hair loss (AZA-related, 2 female cases), muscle cramps (TAC-related, 1 case, which disappeared after dose adjustment), and transient elevation of aminotransferases (4 cases, which disappeared after dose adjustment) during the follow-up. In some early studies of oral medication for MG, TAC, MMF, and AZA were discussed as potentially beneficial for MG, with the greatest benefit being the asteroid-sparing effect (18–20). However, the greatest drawback of the above studies is that the types of antibodies studied were not elucidated. MG is a highly heterogeneous group of autoimmune diseases and the subclass to which the antibodies belong will directly affect the outcome of the study; differences between MuSK-MG and AchR-MG in response to immunomodulatory regimens were discussed by several authors (3–5, 21, 22). Our prospective study was designed to offer a new approach to reducing relapse in Chinese patients with MuSK-MG.

Our study showed that 22/39 (56.4%) patients in the subgroup with IS- experienced a relapse, with a significant decrease in relapse events in the IS+ group (14.3%). The median time to relapse in the IS- the group was 22.5 months, and the median time to relapse could not be calculated for the IS+ group as only 2 relapses occurred. Some previous literature did not discuss steroid monotherapy separately from steroids in combination with IS therapy for MuSK-MG, where the proportion of relapse was 23 to 40% without subgrouping, and the median time to relapse was 22 months (23, 24). The relapse rate in the IS+ group in this study was lower than that in published studies, we speculated that it was due to the following mechanisms: 1) it was related to the low-dose steroid maintenance therapy in this study and 84.9% of the study subjects were unsuccessful in discontinuing steroid at the end of follow-up; 2) the lower MGFA of the enrolled patients compared to other studies, with 13.2% MGFA-V in this study and a maximum of 47% in other similar studies (23, 25).

It is increasingly recognized that MuSK-MG responded well to steroid treatment, and traditional oral non-steroid IS is not a substitute for long-term steroid maintenance therapy (26, 27); our study showed similar results. Although long-term low-dose steroid maintenance therapy cannot reduce relapse, it cannot be excluded that it plays a role in the persistence of non-steroid IS. In our study, there was a higher proportion of steroid therapy in the IS+ group at time 0, and inconsistency between individuals in the way steroids and IS were maintained and reduced, which may lead to bias in our findings.

Previously, it was thought that MuSK antibody titers were significantly correlated with disease severity and that MuSK antibody titers could decrease as patients achieved remission (27). We did not find a correlation between higher antibody titers and the probability of relapse, and there were no significant differences for various factors such as duration of steroid use, the median time to relapse, steroid daily dose at the end of follow-up, or MGC score and MG-ADL at the end of follow-up between different antibody titer subgroups. We speculate that antibodies may be a concomitant manifestation of the active state of the disease.

Our cohort study showed that more than half of MuSK-Patients with MG achieved minimal manifestation (MM) and better at the end of follow-up (Table 1B), which is consistent with the findings in a previous study [PIS-MM and better = 5/21(23.8%]) PIS-I = 13/21(61.9%)] (23). It is worth noting that our study specified that IVIG or methylprednisolone pulse therapy was only applied when the Man G crisis occurred, and no patients had received PLEX or rituximab, which may have led to a more conservative perception of the study results.

Our study has several limitations. Firstly, this is an observational real-world study, randomization was not applied to group patients, and the results could be biased by baseline characteristics. Secondly, the number of patients enrolled is smaller compared to the ideal model, which may introduce additional bias to the study conclusion and subgroup analysis. We should be more cautious in interpreting the results of data analysis, and relevant findings need to be confirmed in future studies involving a larger patient population. Thirdly, we cannot exclude role for the steroid in the effect of IS. Finally, considering the effects of non-steroid IS on women of reproductive age, the pros and cons need to be weighed in practical application.

## Conclusions

Our study adds to the current information available on MuSK-MG treatment, which has been hampered by the small number of studies and many methodological flaws, suggesting that non-steroid IS use is the only factor associated with relapse. In addition, we found that MuSK-MG with a longer duration and a general manifestation (MGFA II and even severer MGFA) may have a better response to treatment with non-steroid IS, which was not reported in previous studies. Furthermore, we reviewed this large cohort at our institution to evaluate the clinical course and long-term outcomes of Chinese MuSK-MG populations. It is hoped that a prospective randomized trial will be available in the future to observe the efficacy and safety of IS in the treatment of MuSK-MG and that the exploration of the specific mechanisms of IS onset will be an important task to be addressed in the future.

## Data Availability Statement

The raw data supporting the conclusions of this article will be made available by the authors, without undue reservation.

## Ethics Statement

The studies involving human participants were reviewed and approved by Ethics Committee of Peking Union Medical College Hospital, Chinese Academy of Medical Sciences, China. The patients/participants provided their written informed consent to participate in this study.

## Author Contributions

YT contributed to drafting and revising the manuscript, study concept and design, acquisition of data, and statistical analysis. LZ contributed to the lab work. YH, KL, JY, and JS contributed to the acquisition of data and interpretation of the data. YG and LC contributed to drafting and revising the manuscript, study concept and design, and interpretation of the data. All authors contributed to the article and approved the submitted version.

## Funding

This work was supported by Beijing Municipal Sciences and Technology Commission (Z181100001718145), National Clinical Cohort Study of Rare Diseases Program (2016YFC0901501), and the Non-profit Central Research Institute Fund of Chinese Academy of Medical Sciences (2019XK320039).

## Conflict of Interest

The authors declare that the research was conducted in the absence of any commercial or financial relationships that could be construed as a potential conflict of interest.

## Publisher's Note

All claims expressed in this article are solely those of the authors and do not necessarily represent those of their affiliated organizations, or those of the publisher, the editors and the reviewers. Any product that may be evaluated in this article, or claim that may be made by its manufacturer, is not guaranteed or endorsed by the publisher.
