# Supplementary material for: Long-Term Efficacy of Non-steroid Immunosuppressive Agents in Anti-Muscle-Specific Kinase Positive Myasthenia Gravis Patients: A Prospective Study
Source: Front Neurol. 2022 Jun 13;13:877895. doi: 10.3389/fneur.2022.877895 (PMC9237788; doi:10.3389/fneur.2022.877895)
Supplement: Supplementary file 1 [file Table_1.DOCX]

| Table_e1 Characteristics of MuSK-MG with different Antibody titers | | | | |
| --- | --- | --- | --- | --- |
| Name | Higher Ab | Lower Ab | Total ^c^ | P value |
| Patients, n | 26 | 24 | 50 | NA |
| Female, n/total | 20/26 | 17/24 | 37/50 | 0.47^a^ |
| Later onset, n/total | 7/26 | 12/24 | 19/50 | 0.15^a^ |
| Disease duration, months, Median (IQR) | 13 (7.75, 51) | 12 (5, 30) | 12 (6, 47) | 0.29^b^ |
| History of relapse, n/total | 10/26 | 5/24 | 20/50 | 0.22^a^ |
| Corticosteroid therapy, n/total | 14/26 | 9/24 | 23/50 | 0.54^a^ |
| History of IS therapy, n/total | 3/26 | 2/24 | 5/50 | 1.00^a^ |
| IVIG within 3 months, n/total | 8/26 | 12/24 | 20/50 | 0.26^a^ |

^a Fisher exact test; b Mann-Whitney U test; c 3 missing data points.^
